# Use of heart rate variability in prediction of clinical deterioration in critically ill hospitalised adults: a systematic review protocol

**DOI:** 10.1136/bmjopen-2026-118185

**Published:** 2026-07-29

**Authors:** Stanley Goodbody, Brian W Johnston, Chloe Lord, Angela Hall, Ingeborg D Welters

**Affiliations:** 1 University of Liverpool Hospitals NHS Foundation Trust, Aintree University Hospital, Liverpool, England, UK; 2 School of Medicine Liverpool, University of Liverpool, Liverpool, England, UK; 3 Institute of Ageing and Chronic Disease, Faculty of Health and Life Sciences, University of Liverpool, Liverpool, England, UK; 4 Liverpool Centre for Cardiovascular Sciences Liverpool, University of Liverpool, Liverpool, England, UK; 5 Intensive Care Unit, Royal Liverpool University Hospital, Liverpool, England, UK; 6 Wirral University Teaching Hospital NHS Foundation Trust, Arrowe Park Hospital, Wirral, England, UK; 7 Library and Knowledge Services, University of Liverpool Hospitals NHS Foundation Trust, Royal Liverpool Hospital, Liverpool, UK

**Keywords:** Mortality, Artificial Intelligence, Physiological Stress, CARDIOLOGY, Adult intensive & critical care

## Abstract

**Abstract:**

**Introduction:**

Heart rate variability (HRV) is a measurement derived from the beat-to-beat variation in heart rate and reflects the complex interaction of physiological systems, including autonomic, endocrine, immune and cardiorespiratory. Reduced HRV has been shown to be associated with adverse outcomes in several disease states. In critical illness, commonly used prognostic tools such as the Acute Physiology and Chronic Health Evaluation II score rely on intermittently collected or rapidly obsolete data and may fail to detect acute deterioration. Continuous, non-invasive HRV monitoring may be able to predict physiological deterioration effectively in critically ill adults. However, its clinical utility remains uncertain due to methodological heterogeneity and a multitude of confounding factors. This systematic review aims to evaluate the prognostic value of HRV and HRV-derived measures for mortality and other clinically significant outcomes in critically ill adults. Furthermore, it will appraise the methods used to measure HRV in this population.

**Methods and analysis:**

This systematic review protocol is reported in accordance with the Preferred Reporting Items for Systematic Reviews and Meta-Analyses (PRISMA) guidelines. Randomised and non-randomised studies involving hospitalised, non-pregnant adults who are critically ill (as defined by author) will be included. Studies must use HRV or HRV-derived measures to predict mortality or other predefined clinically significant outcomes to be included. Searches will be conducted in Embase, MEDLINE, Scopus, Web of Science, American Psychological Association (APA) PsycINFO and the Cochrane Central Register of Controlled Trials, with no date restrictions. Two reviewers will independently perform screening, data extraction and risk of bias assessment—with a third reviewer to resolve conflicts. The primary outcome is all-cause mortality. Secondary outcomes include Intensive Care Unit (ICU) and hospital length of stay, mechanical ventilation and renal replacement therapy requirement and arrhythmia burden. Results will be synthesised narratively, with random-effects meta-analysis undertaken if the data permit. Prognostic effect measures (eg, ORs, HRs and risk ratios) will be extracted according to reported mortality time point, with adjusted estimates prioritised over unadjusted estimates where both are available. Methodological quality and adherence to international HRV measurement standards will be assessed. Risk of bias will be assessed using the Cochrane Risk of Bias 2 tool and the Newcastle-Ottawa Scale for randomised and non-randomised studies, respectively. Certainty of evidence will be evaluated using the Grading of Recommendations Assessment, Development and Evaluation approach.

**Ethics and dissemination:**

Ethical approval is not required, as this review will use only published data. This review will appraise the current literature on HRV as a prognostic marker in the critically ill and audit the data collection methods employed against international standards to give insight into its clinical utility and inform future research. Findings will be disseminated through a peer-reviewed journal publication as well as presentation in the relevant settings.

**Registration:**

PROSPERO CRD420251175808

**STRENGTHS AND LIMITATIONS OF THIS STUDY**

- This study is protocol driven with a systematic and transparent methodology in line with Preferred Reporting Items for Systematic Review and Meta-Analysis Protocols (PRISMA-P) guidance.
- Screening, data extraction and risk of bias assessment will be carried out in duplicate with a view to quantitatively analysing the results with meta-analysis.
- Author defined populations are inherently heterogeneous and may capture a group with non-uniform characteristics, potentially complicating quantitative synthesis.
- Exclusion of non-English papers may generate bias and reduce external validity of findings.

## Introduction

 Heart rate variability (HRV) is a physiological measurement derived from the time difference between the successive normal R-wave peaks on an ECG. Traditionally, it was thought to reflect dynamic modulation of the sinoatrial node (SAN) from the opposing signals of the sympathetic and parasympathetic nervous systems. However, more recent research has described a complex interplay between multiple other biological systems, including endocrine, immune and respiratory as well as effects of the SAN itself.^1^ A reduced HRV is suggested to reflect autonomic imbalance with poor physiological reserve and is therefore associated with pathological states. Foetal HRV monitoring is a long-established tool for recognising foetal distress and bears a strong influence on clinical decision-making.^2^

There is a growing body of literature demonstrating the potential prognostic utility of HRV in various pathological states. Examples include sepsis, adverse cardiovascular events, chronic obstructive pulmonary disease exacerbation and in the postoperative setting.^3–6^ Despite this, its clinical use has not been formally adopted outside of obstetrics.

In critical illness, there are several scoring systems which are used to predict clinical deterioration—namely, the Acute Physiology and Chronic Health Evaluation II (APACHE II) score, the Sequential Organ Failure Assessment score and, to a degree, the National Early Warning Score 2.^7–9^ These composite scores are calculated at intervals using vital signs, patient demographics, laboratory results and use of organ support to predict clinical course. However, the data required for these scores are not always readily available, and periodic measurements may fail to capture acute deterioration. Real-time, non-invasive HRV measurement overcomes these limitations and is suited to the critical care context where patients are often already subject to continuous monitoring.

There are challenges with measuring HRV, particularly in the critically ill patient. Many interventions used in this population can influence the measurement, for example, sympathomimetic medication including epinephrine and norepinephrine, sedative medications and mechanical ventilation.^10^ Furthermore, patient factors can also confound HRV monitoring—examples include chronic diseases such as diabetes, hypertension and heart failure^11–13^ as well as acute pathological states such as arrhythmias and haemodynamic instability.^14^

A further issue in this field of research is that HRV can be recorded in multiple different indexes, usually categorised into time-domain (statistical and geometric), frequency-domain (short term and long term) and non-linear measures (Poincaré analysis, entropy analysis and detrended fluctuation analysis) (table 1). Despite efforts to standardise measurements,^15–17^ there is still a considerable lack of methodological consistency in how HRV data are collected. As a result of this and a failure to adjust for confounding factors, pooling of studies for systematic review and meta-analysis has previously been challenging and ultimately impeded a robust body of evidence to support the use of HRV in critical illness.^14^

**Table 1:** Methods of HRV measurement such as time-domain, frequency-domain and non-linear measurements will be included

Time domain variables	Units	Descriptions
Statistical measures		
SDNN	ms	SD of all normal-to-normal (NN) intervals during the recording
SDANN	ms	SD of the averages of NN intervals in all 5 min sections of the entire recording
RMSSD	ms	Root mean square of the successive differences between adjacent NN intervals
SDNN index	ms	Mean of the SD of NN intervals for all 5 min sections of the recording
SDSD	ms	SD of differences between adjacent NN intervals
NN50 count		Number of successive NN interval pairs differing by more than 50 ms
pNN50	%	NN50 count divided by the total number of all NN intervals
Geometric measures		
HRV triangular index		Integral of RR interval histogram divided by height
TINN	ms	Baseline width of the NN interval histogram
Differential index	ms	Derived from the distribution of differences between successive NN intervals, calculated as the difference between the histogram widths measured at predefined heights
Logarithmic index		The parameter of a negative exponential function that best fits the histogram of absolute differences between consecutive NN intervals
Frequency domain variables	Units	Descriptions
Total power	ms²	Variance of NN intervals across all analysed frequency components
Ultra-low frequency (ULF)	ms²	Very slow oscillations observable in long-term recordings
Very-low frequency (VLF)	ms²	Slow oscillations influenced by long-term regulatory mechanisms
Low frequency (LF)	ms²	Reflects a combination of sympathetic and parasympathetic influences on heart rate modulation
High frequency (HF)	ms²	Reflects parasympathetic (vagal) modulation of heart rate and is closely related to respiratory sinus arrhythmia
LF normalised units	n.u.	LF power expressed relative to total power minus VLF, emphasising the relative contribution of LF oscillations
HF normalised units	n.u.	HF power expressed relative to total power minus VLF, emphasising relative vagal modulation
LF/HF ratio	–	Ratio of LF to HF power, commonly used as an index of sympathovagal balance
Non-linear variables	Units	Descriptions
Poincaré plot analysis—SD1	ms	SD perpendicular to line of identity—reflects short-term HRV complexity
Poincaré plot analysis—SD2	ms	SD along line of identity—reflects long-term variability patterns
SD1/SD2 ratio		Ratio of short- to long-term variability—marker of relative complexity of autonomic modulation
Approximate entropy (ApEn)		Quantifies regularity and unpredictability in time series; higher values indicate less predictability/greater complexity
Sample entropy (SampEn)		Refined entropy measure less dependent on data length—assesses signal irregularity

HRV, heart rate variability; NN50, The count (number) of pairs of adjacent NN intervals that differ by more than 50 milliseconds over the recording period; pNN50, The proportion (percentage) of NN50 divided by the total number of NN intervals; RMSSD, Root Mean Square of Successive Differences; SDANN, Standard Deviation of the Averages of NN intervals; SDNN, Standard Deviation of NN intervals; SDSD, Standard Deviation of Successive Differences; TINN, Triangular Interpolation of the NN interval histogram.

This systematic review aims to evaluate the utility of HRV and HRV-derived measures in predicting mortality and other clinically significant outcomes in critically ill adults. Additionally, the review will identify and critically appraise the methodological approaches used in HRV measurement within the research.

### Specific objectives

To investigate the utility of HRV measurements in predicting mortality and other clinically relevant outcomes in critically ill patients.

### Protocol development

This protocol has been conducted in line with the ‘Preferred Reporting Items for Systematic Review and Meta-Analysis Protocols’ (PRISMA-P), and similarly, the planned systematic review will be undertaken and reported in accordance with ‘Preferred Reporting Items for Systematic Reviews and Meta-Analyses’ (PRISMA). We have registered this review prospectively on PROSPERO register of systematic reviews (registration ID: CRD420251175808).

## Eligibility criteria

### Population

The population of interest is hospitalised, non-pregnant adults who are critically ill. Therefore, studies whose participants do not meet these criteria will be excluded. See table 2 for full inclusion and exclusion criteria.

**Table 2:** Inclusion and exclusion criteria

Inclusion	Exclusion
Hospitalised non-pregnant adults who are critically ill as defined by the author	Aged<18 years
Studies which use HRV measurement or HRV-derived measurements as a predictive marker for clinical deterioration	Participants who are not deemed critically ill by the author
Studies which report any of the following outcomes: mortality, ICU length of stay, hospital length of stay, ICU admission, mechanical ventilation requirement, renal replacement therapy requirement, arrhythmia burden, vasopressor or inotrope requirement	Case reports of less than 10 participants, reviews, conference presentations and editorials
	Studies not published in English
	Studies based in the outpatient or pre-hospital context

HRV, heart rate variability.

In this review, any definition of ‘critical illness’ proposed by the original study authors will be included. We believe this is a justifiable approach due to the lack of a universally accepted definition and reflects the heterogeneity of how critical care is delivered across healthcare settings with varying resource availability and local practices. In the United Kingdom, the Guideline for the Provision of Intensive Care Services (GPICS) uses a practical definition of critical illness as the requirement for level 2 or level 3 care.^18^ Applying the GPICS definition in our inclusion criteria would risk excluding relevant evidence and reduce generalisability. Once screening is complete, author definitions of critical illness will be extracted, reported and scrutinised.

### Intervention

Studies will be included if they use HRV measurements or HRV-derived measurements to predict clinical deterioration. This can include direct measurements of HRV, composite scores and artificial intelligence or machine learning informed models. Methods of HRV measurement such time-domain, frequency-domain and non-linear measurements will be included (table 1).

### Outcomes

The primary outcome will be all-cause mortality during the reported follow-up. Subgroup analysis will be undertaken if the data allow. Subgroups identified may include but are not limited to admission diagnosis, APACHE II score, medical/surgical patients, demographic subgroups and relevant comorbidities.

Secondary outcomes are ICU length of stay, hospital length of stay, need for ICU admission, mechanical ventilation requirement, renal replacement therapy requirement, arrhythmia burden, vasopressor requirement and inotrope requirement (table 2). These were selected as they are clinically relevant outcomes in the context of critical care.

Where reported, prognostic effect measures will be extracted for each outcome, including ORs, HRs, risk ratios and discrimination measures such as the area under the receiver operating characteristic curve or c-statistic. Where both adjusted and unadjusted estimates are reported, adjusted estimates will be prioritised for data extraction and synthesis, with unadjusted estimates recorded for context. As studies are anticipated to report mortality at differing time points (eg, ICU, hospital, 28-day, 90-day or longer-term mortality), the time point of each mortality estimate will be extracted and reported separately. Data will only be pooled in meta-analysis where studies report the same or a sufficiently comparable time point and effect measure, in line with the approach described in the Synthesis and Meta-Analysis section below.

### Study designs

Randomised and non-randomised controlled trials (cohort and observational studies) will be included in this study. Case reports of less than 10 participants, reviews, conference presentations and editorials will be excluded. Studies written in languages other than English will also be excluded.

### Study setting

This review will look at studies set in hospital—thus excluding any out-of-hospital or pre-hospital contexts. The condition in question is critical illness as defined by the author(s).

### Search methods

#### Patient and public involvement

No patient involvement was sought as this is a systematic review.

### Electronic search

In order to conduct the electronic search, the assistance of an experienced librarian (AH) will be sought. Test articles will be reviewed to identify key words and concepts to include as search terms. The initial search will be developed for Embase and subsequently replicated with the database-specific indexing terms and operators for MEDLINE, Scopus, Web of Science, APA PyschINFO and Cochrane Central Register of Controlled Trials. Boolean operators and controlled vocabulary will be used as appropriate. Due to the number of secondary outcomes, terms for the outcomes will not be included in the search. Instead, relevant outcomes will be selected during the screening process. This will also reduce risk of bias due to missing data.

The key concepts to be included in the search criteria are critical illness, intensive care and HRV. A draft search strategy designed for Embase is given below:

1. critically ill patient/
2. Intensive Care Units/ or Critical Care/ or Coronary Care Units/
3. (“intensive care unit” or ICU or “critical care unit” or “high dependency unit” or HDU or “coronary care unit” or CCU or “respiratory high dependency” or “level three care” or “level 3 care”).ab,kf,ti.
4. (“critical illness” or “critically ill” or “critical patient” or “critical condition”).ab,kf,ti.
5. 1 or 2 or 3 or 4
6. heart rate turbulence/ or heart rate variability/
7. (heart rate adj2 variability).ab,kf,ti.
8. HRV.ab,kf,ti.
9. heart rate variabilit*.ab,kf,ti.
10. (RR adj2 variabil*).ab,kf,ti.
11. (RR adj2 interval*).ab,kf,ti.
12. (NN adj2 variabil*).ab,kf,ti.
13. (NN adj2 interval*).ab,kf,ti.
14. Poincare plot.ab,kf,ti.
15. 6 or 7 or 8 or 9 or 10 or 11 or 12 or 13 or 14
16. 5 and 15
17. 16 not (exp infant/ or exp child/ or exp adolescent/)

No geographical or date restriction will be applied, but only studies published in English will be sought.

In addition to the search of electronic databases, further studies may be identified through reviewing the citations and reference lists of relevant articles.

### Timeline

The electronic search will be completed in July 2026. Screening, extraction and risk of bias assessments will then commence and be complete by the end of August 2026. Synthesis, analysis, write-up and submission will be complete by the end of September 2026.

### Methods of review

#### Screening

Articles retrieved from each database search will be uploaded to the online literature review platform Rayyann (Rayyan Systems, Inc., Cambridge, Massachusetts, USA). Here, articles will be combined, and duplicates will be resolved.

Two independent reviewers (SG and CL) will then undertake title and abstract screening of all papers with a third reviewer (BWJ) available to resolve conflicts. All reviewers will be familiar with the predefined inclusion and exclusion criteria. The same process will be repeated for full-text screening: the same two independent reviewers (SG and CL) with a third (BWJ) to resolve disputes. Reasons for full-text exclusion will be recorded to include in the PRISMA flow-chart. If multiple publications present the same study, the one with the most available data will be included.

### Extraction

Data will be extracted from the remaining full-text articles which comply with both the inclusion and exclusion criteria. Two reviewers (SG and CL) will extract data independently within a predefined data collection form with a third reviewer (BWJ) to resolve inconsistencies. Microsoft Excel (Microsoft Corp., Redmond, Washington, USA) will be used to facilitate extraction. The examples listed below are subject to change depending on the data collected.

Key bibliographical characteristics (eg, title, authors, year of publication, source, funding and country) and methodological features (eg, study design including definition of critical illness, study setting and reported outcomes) will be extracted and used to populate the data collection form.

Furthermore, the participant data from each included study will be collected, including basic demographic information (eg, sample size, age, sex distribution and comorbidities) as well as the relevant active disease processes and physiological status.

The outcomes of each study which fulfil the eligibility criteria will be extracted, including the time point and all relevant statistical descriptions of the findings.

Due to the broad methodological variation in HRV assessment, it is important that the details of HRV measurement are extracted for each study. Depending on data availability, this may include type of analytical domain (eg, time domain, frequency domain or non-linear metrics (table 2)), sampling duration, recording device and software, relevant recording conditions (eg, time of day, ventilation status and patient position), use as a stand-alone measure, composite scoring with other parameters or algorithmic metrics including the use of artificial intelligence and machine learning.

### Synthesis and meta-analysis

Separate tables will be generated to summarise the study characteristics, participant data, HRV measurement and results. Descriptive analysis will then be undertaken.

Results will be thematically combined, and narrative synthesis will be performed. Here, the prognostic use of HRV measurement in the context of critical illness can be assessed qualitatively in terms of mortality and the other clinically significant outcomes.

Furthermore, the method of HRV measurement can be appraised. Importantly, we will assess study adherence to the European Society of Cardiology guidelines on HRV measurement.^15^

Provided an adequate number of studies (eg, >2) that are sufficiently similar in outcome and effect measure are identified, meta-analysis will be performed using a random effects model due to the expected study heterogeneity. Cochrane Review Manager will be used for statistical analysis where required. Statistical heterogeneity will be assessed using the I² statistic. An I² value of <75% will be considered low to moderate heterogeneity—but not used as a strict inclusion criteria. If heterogeneity is considered too great, narrative synthesis will be undertaken in the place of meta-analysis.

### Risk of bias and quality assessment

Risk of bias will be assessed independently by two reviewers (SG+CL), and any disagreements will be resolved through discussion with a third reviewer (BWJ). Cochrane Risk of Bias 2 and Newcastle Ottawa Score will be employed for randomised controlled trials and observational studies, respectively. Through assessment of multiple domains, a judgement will be made of the potential for bias in each included study. Risk of bias analysis will be used to inform interpretation of results and not used in isolation to exclude studies.

To reduce reporting bias from missing data, a thorough search through multiple databases will be performed as described previously. Furthermore, protocols and supplementary materials will be searched to identify any unreported outcomes to qualitatively assess reporting bias. If >9 studies are pooled and meta-analysis is performed, bias due to missing results can be quantitatively assessed using funnel plots and Eggar’s test.

The Grading of Recommendations Assessment, Development and Evaluation approach will be used to assess certainty of findings.

## Ethics and dissemination

Ethical approval is not required, as this review will use only published data.

Using a systematic approach, this review will summarise and appraise the current literature surrounding HRV as a prognostic marker in critical illness focusing on clinically relevant outcomes. Furthermore, we will audit the data collection methods employed in these studies and report adherence to international guidance. In this way, we aim to give insight into the clinical utility of HRV in this context and inform potential areas of future research. Findings will be disseminated through a peer-reviewed journal publication as well as presentation in the relevant settings.

## Data Availability

No data are available.
