# Supplementary material for: Use of heart rate variability in prediction of clinical deterioration in critically ill hospitalised adults: a systematic review protocol
Source: BMJ Open. 2026 Jul 29;16(7):e118185. doi: 10.1136/bmjopen-2026-118185 (PMC13422795; doi:10.1136/bmjopen-2026-118185)
Supplement: Supplementary file 1 [file bmjopen-2026-118185.reviewer_comments.pdf]

PEER REVIEW HISTORY

BMJ Open publishes all reviews undertaken for accepted manuscripts. Reviewers are asked to complete a checklist review form and are provided with free text boxes to elaborate on their assessment. These free text comments are reproduced below.

ARTICLE DETAILS

Title (Provisional)

'The Use of Heart Rate Variability in Prediction of Clinical Deterioration in Critically Ill Hospitalised Adults' -A Systematic Review Protocol

Authors

Goodbody, Stanley; Johnston, Brian W; Lord, Chloe; Hall, Angela; Welters, Ingeborg D

VERSION 1 - REVIEW

**Reviewer**	**1**
**Name**	**D'Ascenzo, Fabrizio**
**Affiliation**	**San Giovanni Battista, Molinette**
**Date**	**24-May-2026**
**COI**	**None**

---

interesting paper. Some issues need to be addressed.

his protocol outlines a systematic review evaluating heart rate variability (HRV) as a predictor of clinical deterioration in critically ill hospitalized adults.

The review will investigate whether HRV can predict mortality and other clinically relevant outcomes such as ICU admission, mechanical ventilation, and vasopressor requirement.

Both randomized and observational studies involving critically ill adult inpatients will be included, while pediatric, outpatient, and non-English studies will be excluded.

Multiple HRV assessment methods will be analyzed, including time-domain, frequency-domain, and non-linear HRV measures.

The review will follow PRISMA-P methodology, with independent screening, data extraction, and risk-of-bias assessment performed by two reviewers.

Findings will help clarify the clinical utility of continuous HRV monitoring in critical care and identify methodological limitations for future research.

**Reviewer**	**2**

Name **Krige, Anton**

Affiliation **Royal Blackburn Teaching Hospital, Anaesthesia and Critical Care**

Date **24-Jun-2026**

COI **None**

This is an important systematic review and is required as a baseline before further prospective research in this area.

Overall recommendation

This is a relevant and well-designed systematic review protocol addressing an important question in critical care prognostication. The manuscript is suitable for publication after minor revision, mainly to provide search strategy details, handling of prognostic effect measures, and some wording/typographical issues.

1. Is the research question or study objective clearly defined?
    a. The research aim is clearly stated in the abstract and introduction: to evaluate the prognostic value of HRV and HRV-derived measures for mortality and other clinically significant outcomes in critically ill adults, and to appraise HRV measurement methods.
2. Is the abstract accurate, balanced and complete?
    a. The abstract appropriately reflects the manuscript as a protocol and summarises the rationale, eligibility criteria, databases, duplicate review process, risk-of-bias assessment, and planned synthesis. It would be stronger if it briefly listed the main secondary outcomes.
3. Is the study design appropriate to answer the research question?
    a. A systematic review of randomised and non-randomised studies is appropriate for this prognostic question, especially given the likely predominance of observational HRV literature. The planned use of narrative synthesis with random-effects meta-analysis only where data are sufficiently similar is also appropriate.
4. Are the methods described sufficiently to allow the study to be repeated?
    a. The eligibility criteria, databases, duplicate screening, duplicate data extraction, and planned quality appraisal are generally well described. Reproducibility would improve by including a full draft search strategy for at least one database, clarifying the truncated sentence on language restriction, and pre-specifying how different mortality time points and different prognostic effect measures will be handled.
5. Are the outcomes clearly defined?
    a. The primary outcome is clearly stated as all-cause mortality during reported follow-up, and the secondary outcomes are explicitly listed. Clarification would help on how different mortality time points will be grouped and how "arrhythmia burden" will be defined if studies use heterogeneous metrics.
6. If statistics are used are they appropriate and described fully?
    a. The planned use of random-effects meta-analysis, I^2 to assess heterogeneity, funnel plots/Egger's test where appropriate, and GRADE is reasonable. However, the protocol should better specify which prognostic effect measures will be extracted or prioritised, how adjusted versus unadjusted estimates will

be handled, and what degree of clinical/methodological similarity will be required for pooling.

7. Are the references up-to-date and appropriate?
    a. The references are appropriate for this protocol and include foundational HRV standards and more recent methodological and disease-specific literature, including critical care–relevant sources and current GPICS guidance.
8. Are the study limitations discussed adequately?
    a. The manuscript acknowledges important anticipated limitations, including heterogeneity from author-defined critical illness, expected predominance of observational studies, and inconsistent outcome measures. It would be helpful also to acknowledge possible language bias from English-only inclusion.
9. Is the supplementary reporting complete (e.g. trial registration; funding details; CONSORT, STROBE or PRISMA checklist)?
    a. PROSPERO registration is provided, and the protocol states adherence to PRISMA-P, with PRISMA planned for the final review. Funding, competing interests, author contributions, and ethics statements are also included. Submission of a PRISMA-P checklist and full search strategy should be ensured if not already provided separately.
10. Is the standard of written English acceptable for publication?
    a. The manuscript is generally clear and readable. A small number of typographical and grammatical errors should be corrected, such as “critical ill” instead of “critically ill,” the incomplete sentence regarding English-language studies, spelling consistency, and minor formatting issues. See some of the issues noted listed at the bottom of Minor commnents.

Major comments

1. Improve reproducibility of the search strategy. The manuscript names the databases and the key concepts, but it does not provide a full draft strategy for any database. Including the full MEDLINE or Embase strategy, ideally in a supplement, would materially improve transparency and reproducibility.
2. Clarify handling of prognostic effect measures. Because this is a prognostic review, the protocol should specify which summary measures will be extracted and prioritised, such as odds ratios, hazard ratios, risk ratios, discrimination metrics such as AUC/c-statistics, or calibration measures if reported. It should also clarify whether adjusted estimates will be prioritised over unadjusted estimates.
3. Clarify how heterogeneous outcome time points will be synthesised. The primary outcome is all-cause mortality during reported follow-up, but studies may report ICU mortality, hospital mortality, 28-day mortality, or longer-term mortality. Pre-specifying how these will be grouped or analysed separately would strengthen the protocol.

Minor comments

1. The sentence in the search methods section - Line 169/170 - appears incomplete: “only published studies which are written in will be sought.”
2. Consider explicitly listing the principal secondary outcomes alongside mortality, e.g. critical care length of stay
3. Consider acknowledging English-language restriction as a potential limitation and source of language bias.
4. Minor typographical issues should be corrected, including some grammatical errors and consistency of spelling/punctuation. Some e.g.’s:

- Line 15 – systems, including
- Line 35 - if the data permit.
- Line 38 - non-randomised studies, respectively

- Line 42 suggested wording - Ethical approval is not required, as this review will use only published data.
- Line 45 - disseminated through a peer-reviewed journal
- Line 56 - biological systems, including endocrine
- particularly in the *critically* ill patient.
- Line 133 - Subgroup analysis will be undertaken if the data allows it. Subgroups identified may include, but are not limited to the admission..
- etc

VERSION 1 - AUTHOR RESPONSE

Response to Reviewer Comments

We thank the reviewer for their careful and constructive review of our protocol. We are pleased that the reviewer considers the manuscript suitable for publication after minor revision. Below we respond to each point in turn; reviewer comments are shown in italics-equivalent plain text preceded by "Reviewer:", followed by our response. Corresponding changes have been made to the manuscript, with new or amended text shown in red in the marked copy.

Overall recommendation

Reviewer: This is a relevant and well-designed systematic review protocol addressing an important question in critical care prognostication. The manuscript is suitable for publication after minor revision, mainly to provide search strategy details, handling of prognostic effect measures, and some wording/typographical issues.

Response: We thank the reviewer for this positive assessment. We have addressed each of the points raised below, including providing a full draft search strategy, clarifying the handling of prognostic effect measures and heterogeneous mortality time points, and correcting the wording and typographical issues identified.

1. Is the research question or study objective clearly defined?

Reviewer: The research aim is clearly stated in the abstract and introduction.

Response: We thank the reviewer for this comment; no changes were requested here.

2. Is the abstract accurate, balanced and complete?

Reviewer: The abstract appropriately reflects the manuscript as a protocol. It would be stronger if it briefly listed the main secondary outcomes.

Response: We agree and have amended the Methods and analysis section of the abstract to explicitly list the principal secondary outcomes (ICU and hospital length of stay, mechanical ventilation and renal replacement therapy requirement, and arrhythmia burden), alongside a brief statement on how prognostic effect measures and mortality time points will be handled.

3. Is the study design appropriate to answer the research question?

Reviewer: A systematic review of randomised and non-randomised studies is appropriate for this prognostic question. The planned use of narrative synthesis with random-effects meta-analysis only where data are sufficiently similar is also appropriate.

Response: We thank the reviewer for this comment; no changes were requested here.

4. Are the methods described sufficiently to allow the study to be repeated?

Reviewer: Reproducibility would improve by including a full draft search strategy for at least one database, clarifying the truncated sentence on language restriction, and pre-specifying how different mortality time points and different prognostic effect measures will be handled.

Response: A full draft Embase search strategy is provided in the Search Methods section. We have corrected the truncated sentence, which now reads: "No geographical or date restriction will be applied, but only studies published in English will be sought." We have also added a new paragraph to the Outcomes section specifying that prognostic effect measures (odds ratios, hazard ratios, risk ratios, and discrimination measures such as AUC/c-statistic) will be extracted, with adjusted estimates prioritised over unadjusted estimates where both are reported, and that mortality will be extracted and reported according to the time point described (e.g. ICU, hospital, 28-day, 90-day or longer-term), with pooling restricted to studies reporting a sufficiently comparable time point and effect measure.

5. Are the outcomes clearly defined?

Reviewer: The primary and secondary outcomes are explicitly listed. Clarification would help on how different mortality time points will be grouped and how "arrhythmia burden" will be defined if studies use heterogeneous metrics.

Response: As above, we have clarified the handling of heterogeneous mortality time points in the Outcomes section. Regarding arrhythmia burden, the specific metric used by each study (e.g. incidence of new-onset arrhythmia, arrhythmia-free time, or a validated burden score) will be extracted as reported by study authors and described narratively; we have not imposed a single definition a priori given the range of ways this outcome is reported in the underlying literature, consistent with our approach to author-defined critical illness.

6. If statistics are used are they appropriate and described fully?

Reviewer: The planned statistical approach is reasonable, but the protocol should better specify which prognostic effect measures will be extracted or prioritised, how adjusted versus unadjusted estimates will be handled, and what degree of clinical/methodological similarity will be required for pooling.

Response: We have addressed the prioritisation of adjusted over unadjusted estimates and the range of effect measures to be extracted in the new Outcomes section paragraph described above. The degree of similarity required for pooling is addressed in the existing Synthesis and Meta-Analysis section, which specifies that meta-analysis will only be undertaken where an adequate number of studies (>2) are sufficiently similar in outcome and effect measure, using a random-effects model given the anticipated heterogeneity; we have cross-referenced this section from the new Outcomes paragraph for clarity.

7. Are the references up-to-date and appropriate?

Reviewer: The references are appropriate for this protocol.

Response: We thank the reviewer for this comment; no changes were requested here.

8. Are the study limitations discussed adequately?

Reviewer: It would be helpful also to acknowledge possible language bias from English-only inclusion.

Response: This is already acknowledged in the Strengths and Limitations of this Study section of the abstract ("Exclusion of non-English papers may generate bias and reduce external validity of findings"); no further change was required.

9. Is the supplementary reporting complete?

Reviewer: PROSPERO registration and PRISMA-P adherence are stated. Submission of a PRISMA-P checklist and full search strategy should be ensured if not already provided separately.

Response: The full draft Embase search strategy is now included in the main text of the Search Methods section, and a completed PRISMA-P checklist has been submitted alongside this response as a supplementary file.

10. Is the standard of written English acceptable for publication?

Reviewer: A small number of typographical and grammatical errors should be corrected.

Response: All typographical and grammatical issues identified by the reviewer have been corrected; see our response to the Minor comments below for details of each change.

Major comments

1. Improve reproducibility of the search strategy.

Reviewer: The manuscript names the databases and the key concepts, but it does not provide a full draft strategy for any database. Including the full MEDLINE or Embase strategy, ideally in a supplement, would materially improve transparency and reproducibility.

Response: A full 17-line draft Embase search strategy is provided in the Search Methods section of the manuscript, covering the critical illness and HRV concept blocks and the exclusion of paediatric records. We have retained this within the main text, as we believe this aids transparency for readers, and have also ensured it is reflected in the accompanying PRISMA-P checklist.

2. Clarify handling of prognostic effect measures.

Reviewer: The protocol should specify which summary measures will be extracted and prioritised, such as odds ratios, hazard ratios, risk ratios, discrimination metrics such as AUC/c-statistics, or calibration measures if reported. It should also clarify whether adjusted estimates will be prioritised over unadjusted estimates.

Response: We have added a new paragraph to the Outcomes section stating that odds ratios, hazard ratios, risk ratios and discrimination measures (AUC/c-statistic) will be extracted where reported, and that adjusted estimates will be prioritised over unadjusted estimates for data extraction and synthesis where both are available, with unadjusted estimates recorded for context. This is also now reflected in the abstract.

3. Clarify how heterogeneous outcome time points will be synthesised.

Reviewer: Studies may report ICU mortality, hospital mortality, 28-day mortality, or longer-term mortality. Pre-specifying how these will be grouped or analysed separately would strengthen the protocol.

Response: We have specified in the new Outcomes section paragraph that mortality will be extracted and reported according to the time point described by study authors (e.g. ICU, hospital, 28-day, 90-day or longer-term), that these will be reported separately, and that pooling in meta-analysis will only occur where studies report the same or a sufficiently comparable time point and effect measure.

Minor comments

1. Incomplete sentence at Line 169/170.

Reviewer: "only published studies which are written in will be sought."

Response: Corrected to: "No geographical or date restriction will be applied, but only studies published in English will be sought."

2. List the principal secondary outcomes alongside mortality.

Response: The abstract now explicitly lists the principal secondary outcomes (ICU and hospital length of stay, mechanical ventilation and renal replacement therapy requirement, and arrhythmia burden), in addition to the full list already provided in the main Outcomes section of the manuscript.

3. Acknowledge English-language restriction as a limitation.

Response: This is already stated in the Strengths and Limitations of this Study section of the abstract; no further change was required.

4. Minor typographical issues.

Response: All of the specific examples identified by the reviewer have been corrected, as follows:

- Line 15: "physiological systems including autonomic..." corrected to "physiological systems, including autonomic..."

- Line 35: "if the data permits" corrected to "if the data permit"

- Line 38: "non-randomised studies respectively" corrected to "non-randomised studies, respectively" (corrected at both occurrences in the manuscript)

- Line 42: "Ethical approval is not required as this review will use data from published studies only" corrected to "Ethical approval is not required, as this review will use only published data" (corrected at both occurrences in the manuscript)

- Line 45: "disseminated through peer-reviewed journal" corrected to "disseminated through a peer-reviewed journal" (corrected at both occurrences in the manuscript)

- Line 56: "biological systems including endocrine" corrected to "biological systems, including endocrine"

- "particularly in the critical ill patient" corrected to "particularly in the critically ill patient"

- Line 133: "Subgroup analysis will be undertaken if the data allows it. Subgroups identified may include, but are not limited to the admission.." corrected to "Subgroup analysis will be undertaken if the data allow. Subgroups identified may include, but are not limited to: admission diagnosis..."

We have also re-checked the manuscript throughout for consistency of spelling and punctuation.
